# Detection of Successful and Unsuccessful Pregnancies in Mice within Hours of Pairing through Frequency Analysis of High Temporal Resolution Core Body Temperature Data

**DOI:** 10.1371/journal.pone.0160127

**Published:** 2016-07-28

**Authors:** Benjamin L. Smarr, Irving Zucker, Lance J. Kriegsfeld

**Affiliations:** 1 Department of Psychology, University of California, Berkeley, United States of America; 2 Department of Integrative Biology, University of California, Berkeley, United States of America; 3 The Helen Wills Neuroscience Institute, University of California, Berkeley, United States of America; Kent State University, UNITED STATES

## Abstract

Many controllable factors negatively impact fetal development, underscoring the importance of early pregnancy detection and identification of events that reliably predict potential complications. Clinically, core body temperature (CBT) is used to aid family planning and pregnancy detection. However, such temperature data typically are gathered in single, daily measurements. In animal studies, interventions or cell/tissue harvesting at defined stages of fetal development are arduous, requiring timed mating by trained observers. The value of continuous temperature measurements remains largely unexplored, but the advent of small, inexpensive, and increasingly ubiquitous, accurate sensor devices makes continuous measures feasible. Here, using a mouse model, we show that continuous, 1-min resolution CBT measurements reliably allow for the earliest and most accurate detection of pregnancy (100%, within 14 h of initial pairing), without requiring interaction with the animal for data collection. This method also reveals a subset of females that exhibit a pregnancy-like response following pairing that persists for a variable number of days. Application of wavelet analysis that permits frequency analysis while preserving temporal resolution, uncovers significant differences in ultradian frequencies of CBT; these rhythms are significantly increased in the 12 h after the day of pairing for pregnancies carried to term compared to apparent pregnancies that failed. High temporal resolution CBT and wavelet analysis permit strikingly early detection and separation of successful pregnancies and pregnancy-like events.

## Introduction

Early *in-utero* exposure to myriad environmental insults leads to abnormal fetal development or the termination of pregnancy, underscoring the importance of accurate and convenient early detection strategies. Additionally, in animal models, reliable confirmation of pregnancy following mating permits interventions at defined stages of gestation, including harvesting of age-specific maternal and fetal tissues for stem-cell research. Presently there are few ways of detecting pregnancy prior to implantation (day 3.5–5 of gestation in mice). Increases in body mass, abdominal palpation, or changes in sex steroid concentrations cannot distinguish pregnant from non-pregnant mice prior to 7 days of gestation [1–3], with ultrasound detection possible at 4.5 days [4]. Although changes in progesterone concentrations can be an accurate indicator on the first day of gestation [5], such assessments are invasive, requiring anesthetization and blood withdrawal, both of which may disrupt the pregnancy.

The presence of vaginal plugs deposited during ejaculation is the most commonly used non-invasive marker of gestation onset; vaginal plugs, however, can be poorly formed or dislodged and their presence does not guarantee that a female is pregnant. In outbred CD-1 mice, for example, approximately 70–75% of females exhibiting a vaginal plug have been successfully impregnated; this value decreases to < 50% in inbred females (reviewed in [6]). Depending on housing and other environmental factors, pregnancy rates can range from 33% to 85% in C57BL/6J females exhibiting vaginal plugs [6]. Inspection for vaginal plugs involves disturbing and handling the animal, a stressor that can disrupt the pregnancy prior to implantation.

Continuous recordings of body temperature have been used to stage estrus in rodents [7,8]. A number of hormones, including sex hormones, are known to affect CBT [7–12]. Single time measurements of CBT are also commonly used to detect ovulation and pregnancy in humans, with an increase in basal (trough) body temperature occurring for most individuals in the early morning [13], a change that can still be detected to some degree immediately upon wakening. However, the usefulness of these measures is limited by a lack of resolution and an abundance of signal noise. We hypothesized that higher temporal resolution data would eliminate much of this uncertainty, and allow more rapid and more accurate detection of pregnancy onset. Here we report that continuous monitoring of core body temperature (CBT) of female mice allows pregnancy detection within 14 h of pairing, providing by far the earliest, non-disruptive detection of successful impregnation. Furthermore, this approach allows detection of apparent pregnancies that do not come to term, that would otherwise not be detected by standard handling or observation, providing a potential source of dams for the study of implantation failure, pseudopregnancy, and miscarriage. These apparent pregnancies can be separated from those that will come to term by frequency analysis of high temporal resolution CBT data, in the first 12 h after the day pairing. Together, these findings establish that continuous, high temporal resolution CBT recordings provide a uniquely rapid, accurate, and non-disruptive means of detecting pregnancies and pregnancy outcomes in mice.

## Materials and Methods

**Fig 1:**
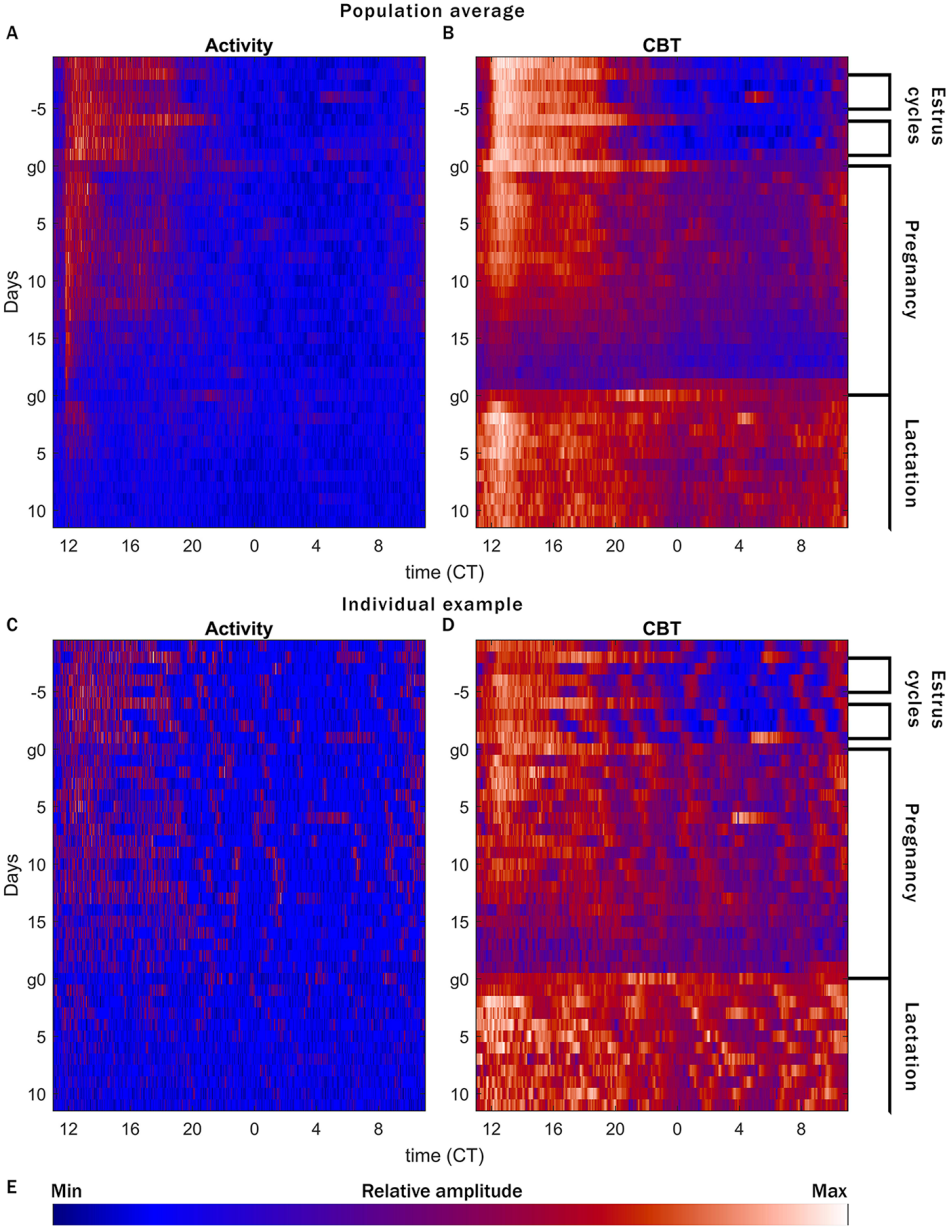
Comparison of CBT and LA data. CBT and LA both reveal changes in female reproduction, but CBT contains more information, especially during times of low LA. Raster plots of relative LA (A, C) or CBT (B, D) counts per minute (color map in E), arranged by ZT (12 = lights off: light:dark bars at the top of each plot indicate the light cycle), with sequential days stacked from top to bottom. Median of 12 mice (A, B) and an individual example mouse (C, D). Patterns matching expectations for estrus—prolonged peak of CBT every 4 days—are apparent in the top 9 rows, more easily detected in CBT than LA. Mice are paired for a single 17 h window encompassing the dark phase of day g0. Birth times are confirmed by twice-daily inspections following g18, and occur on the same day in all pregnancies. After birth, pups remain with the dam until weaning (not shown). Bright spots every 7 days (D) reflect cage changes. Color map (E) shows relative amplitude of LA and CBT. *N*.*B*. the strong ~3h UR in the individual CBT plot (D), which is largely washed out in the median (B).

Data were analyzed from 24 pregnancies in 22 female BALB/c mice (Jackson Labs, Bar Harbor, ME) in accordance with procedures approved by the Animal Care and Use Committee at UC Berkeley and in conformance with principles enunciated in the NIH Guide for the care and use of laboratory animals. Animals were maintained under 12:12 LD cycle of ~400 lux (light) to <1 lux red light (darkness), with lights on from 6am to 6pm. Food and water were available *ad libitum*. Mice CBT profiles were utilized to monitor estrous cycles and mice were paired on the day of apparent estrus (Fig 1) from 2 h before lights-off (4pm) to 3 h after lights-on the following day (9am; 17 h total). An age-matched male was introduced to the female’s home cage for the 17 h duration. Vaginal plugs were not monitored the day after pairing; removal of the male was the only disturbance to the females. All females were nulliparous at the time of pairing, except for 3 mice that were paired twice. Both pregnancies for these mice are described.

### Data acquisition

Data were gathered with Mini Mitter G2 E-Mitter implants for locomotor activity (LA) and CBT (Starr Life Sciences Corp., Oakmont, PA). G2 E-Mitters were implanted in the intraperitoneal cavity under isoflurane anesthesia. Post-operative analgesia was achieved by subcutaneous injections of 0.03 mg/kg buprenorphine in saline, every 12 h for 2 d post-surgery. E-mitters were sutured to the ventral muscle wall to maintain consistent core temperature measurements. Recordings began immediately, but data collected for the first week post-surgery were not included in the analysis. Recordings were continuous at 1-min resolution for LA and CBT. All mice were between 7 and 10 wk of age at the time of implant surgery and were handled once/wk across recordings at the time of cage changes, but otherwise were left undisturbed in single housing.

### Data correction

For CBT records per mouse, all zero or missing values were set equal to the mean of all non-zero, non-missing values for that animal. For LA, zeros were left uncorrected, but missing values were set equal to the mean of all non-missing values. The output from the G2 implant is in the form of °C for CBT and counts per unit time (here, 1 min) for LA.

### Analysis

**Fig 2:**
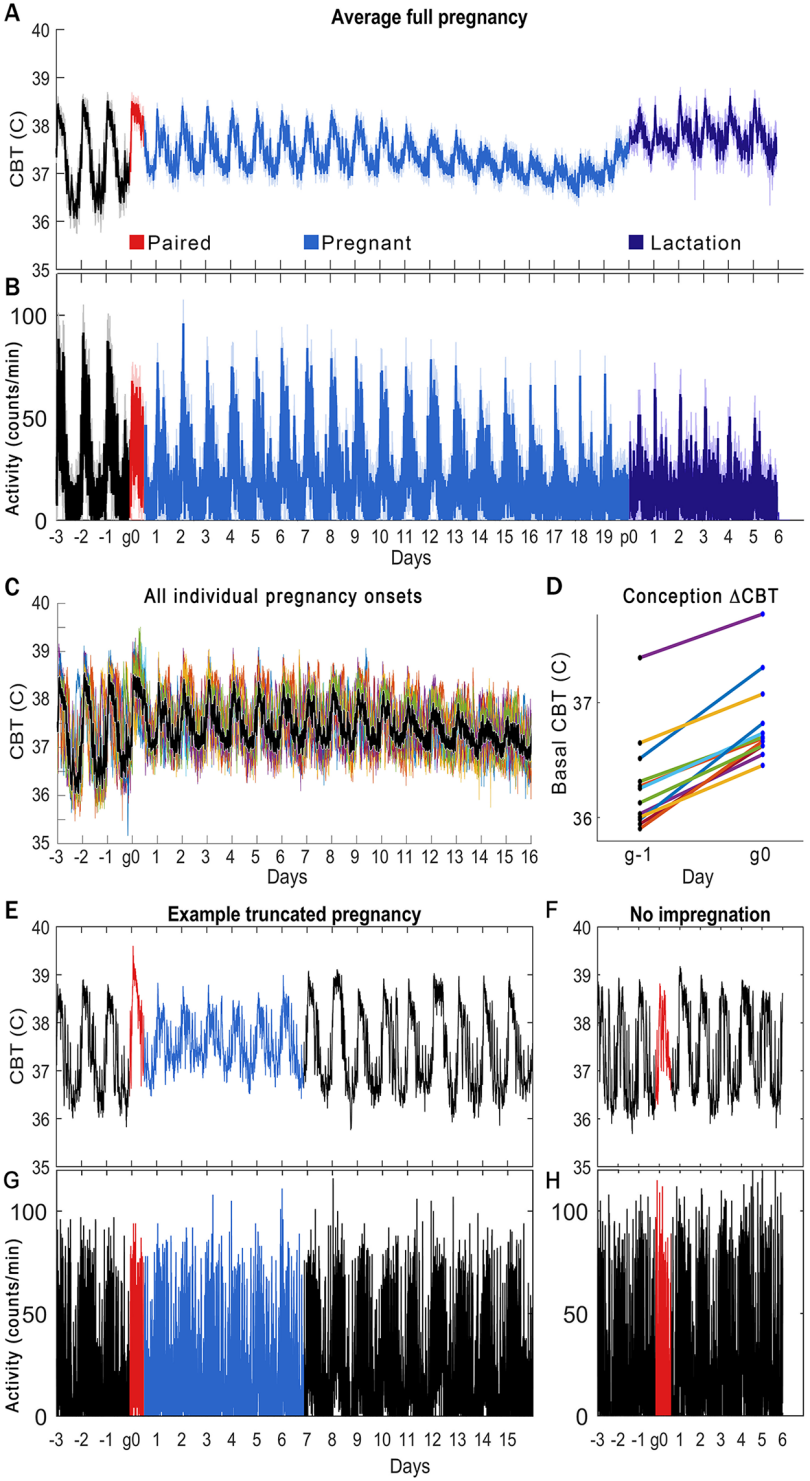
Quantification of CBT changes across pregnancies. CBT changes are readily apparent at pregnancy onset and parturition, and permit detection of pregnancies that will not come to term, and would otherwise be undetected. Time window of pairing is red, pregnancy is blue, and lactation is purple (A, B, E:H). Median +/- standard deviation of 12 pregnancies (A: CBT, B: LA), aligned by the day of pairing (“g0”). Pregnancy onset is detectable in 100% of individuals that come to term as a rise in rest-phase CBT in the first rest-phase following pairing. Eventually decreases in daily LA become detectable (B), but not as quickly as CBT, and with more inter-individual variability. All mice that come to term show this change in CBT. Individual CBT profiles (C) aligned by median of the 3 days before pairing show conservation of shape change in early pregnancy. Individual rest-phase basal CBT on the nights before (g-1) and after (g0) pairing (D) show similar change in all individuals. Not all mice paired show a change in CBT following pairing (F, H), and not all that manifest the initial rise in basal CBT come to term, as exemplified by a mouse showing an initial rise in basal CBT, but returning to apparent 4-day cycling patterns (seen in Fig 1B and 1D, at top) after 7 d of pregnancy-like CBT rhythms (E, G). This number of days varies by individual (data not shown). Note that the pregnancies not carried to term, or pregnancy-like events, are not clearly discernable from the LA profiles.

Data (S1 Dataset) were analyzed and plotted using Matlab 2015b and 2016a in conjunction with in-house code for wavelet decomposition (S1 Code) modified from the “Jlab” toolbox and from code developed by Dr. Tanya Leise [14], using the morse wavelet [15] (β = 5, γ = 3). For statistical comparisons of populations, Wilcoxon rank sum tests were applied to avoid any assumptions about normality for any distribution. Non-parametric Kruskal-Wallis tests replaced ANOVAs for the same reason. In Fig 2D, “basal CBT” for each individual is calculated as the median of the 60 coldest minutes during the light phase for each day. For all tests, pregnancy conditions were treated as independent, and all other data as dependent variables.

### Presentation

A color map for wavelets was developed in house to provide red-green colorblind-compliant uniform contrast across the range of data with the exception of the two extremes (highest and lowest 10%), which are brightened to highlight extreme high and low values. Figures were formatted in Microsoft PowerPoint 2013 and Adobe Photoshop CS6.

## Results

### Rapid Pregnancy Detection

Continuous monitoring of CBT and LA across estrous cycles and pregnancy reveals major changes in reproductive state that appear more clearly in CBT than in LA (Fig 1). While LA can impact CBT, many of the most easily recognized changes in CBT occur during the rest phase, when LA is low. When LA and CBT data are directly compared, the differences become visually apparent. Days of estrus are visible in averaged LA, but are not obvious for individual mice (Fig 1A and 1C). In CBT the days of estrus appear clearly, largely due to the extension of the plateau in CBT beyond the daily active phase.

The daily CBT profile reveals changes at the onset of pregnancy and again at parturition (Fig 1). The median daily range before pairing was 2.85°C (standard deviation +/- 0.27°C); after pairing, CBT remained relatively elevated during the light phase (median daily range shrinks to 1.98 +/- 0.25°C on the day after pairing, *p* = 6.01x10^-5^), rendering pregnancy detectable within 14 h from initial pairing (i.e., the rest phase after initial pairing at 2 h before the onset of the 12 h active, dark phase). This is clear both in the population average, and for individual mice (Fig 1B and 1D “Pregnancy”). LA exhibited no such pattern. Though a gradual decrease in active time is apparent in the population average (Fig 1A), this pattern is not as marked as for CBT, nor consistently apparent for individual mice (Fig 1C). At parturition, LA decreased substantially, presumably due to increased time nursing young. However, as with pregnancy, CBT changed markedly on the day of parturition, with the transition from pregnancy to lactation preceded by a rise in CBT the day prior to birth (structural analysis not shown here). This pattern was maintained for the population average and individuals (Fig 1B and 1D “Lactation”).

Linear comparison of LA and CBT data also demonstrate the rapid and precise detection of pregnancy onset through CBT (Fig 2A and 2B, “g0”). The rise in basal CBT is apparent from the first rest period following pairing (Fig 2, “g0”), and shows stereotyped progression through parturition (“p0”) and lactation. This increase occurred in all mice that came to term (Fig 2C; all individuals’ profiles are aligned to the group median of the first three days so that the shape and absolute amount of change is illustrated), with an average within-individual increase of 0.49 +/- 0.17°C (Fig 2D). This change allows for separating mice that do not deliver a litter into two groups: those that show no detectable change in CBT pattern (Fig 2F), and those that appear to initiate but later terminate pregnancy (Fig 2E). No comparable separation was found in the LA data (Fig 2G and 2H).

**Fig 3:**
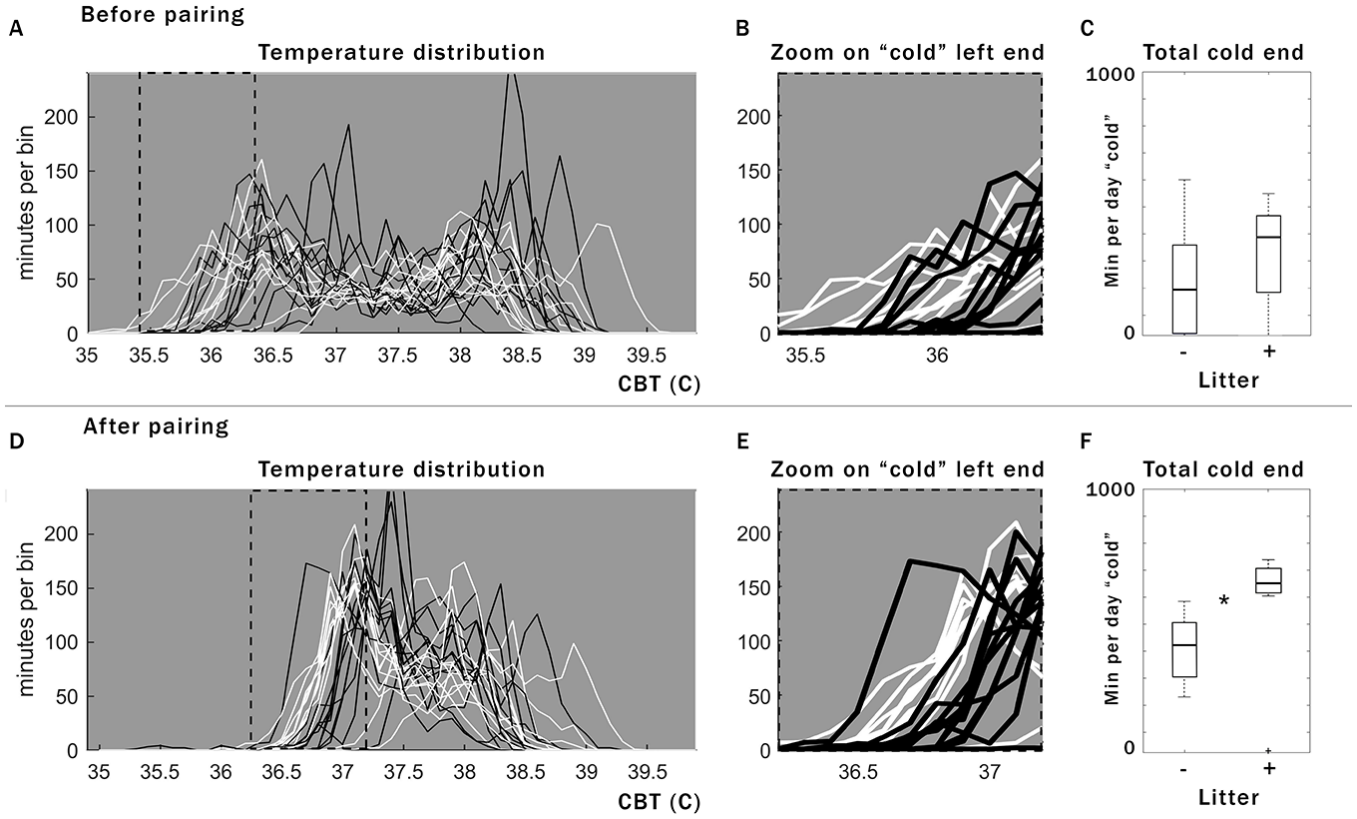
Frequency analysis of daily CBT identifies pregnancy outcome within the first 48 h after pairing. Histogram of daily min per temperature (binned to 0.1 degrees C). For 3 d before pairing (A), no obvious differences distinguish the two populations of ultimately successful pregnancies (white) from those that will show elevated basal CBT after mating, but not come to term (black). The same mice on the second day after pairing (D) illustrate separation of the two populations in the left tail of the histogram (zoom in E and B matched to dotted boxes in A and D). Box plot of the area under the curve shown in the zoom (C for B, F for E) reveals no significant difference before mating, (C) but a significant separation following pairing (asterisk, F). Boxplots shown include outliers, but statistics show additional improvement when outliers are excluded (see Results).

The rise in basal CBT was further analyzed by frequency analysis of daily values, as the distribution of temperatures across the day shrinks to a smaller range than before pairing (Fig 3A and 3D). This frequency analysis identifies 2 outliers in both groups of mice with pregnancy-like responses in CBT (those that eventually come to term, and those that did not). Three of these four individuals show compressed temperature distributions with higher-temperature frequencies similar to the other mice, but no temperature values below 36°C. These patterns are readily apparent in Fig 3D, with CBT rising to the right of the dotted box. Although these females exhibit a rightward shift in their temperature distributions upon pairing similar to the other mice, their distribution is already compressed before pairing. The fourth outlier (Fig 3D and 3E), did not come to term, but exhibits more low-temperature min than any other individual after pairing.

### Early Indicators of Pregnancy Outcome

Frequency analysis allows early separation of apparent pregnancies that are unsuccessful from those that will come to term. In the second day after pairing, the daily CBT distributions reveal a significant shift in the “cold” left tail of the temperature-frequency distribution (Fig 3), with ultimately-successful pregnancies characterized by significantly more min/day of relatively low CBT than for apparent pregnancies which subsequently terminated prematurely (Fig 3D, 3E and 3F; *p* = 0.03 on day 2 post pairing with outliers, 1.84x10^-4^ without outliers). Comparison of average CBT without using frequency analysis (i.e., means of daily, dark phase, or light phase CBT) does not allow this separation. Temperature distributions did not differ significantly before pairing when including the outliers (Fig 3A, 3B and 3C; *p* = 0.13 before pairing with outliers) though a difference emerged when outliers were excluded (*p* = 0.009), largely due to 2 of 12 mice (visible in Fig 3B as the two top white lines). Therefore, for most pregnancies, the post-pairing leftward-shift in CBT distribution does not appear to reflect the mouse’s condition prior to mating, but physiological difference in response to mating; alternatively, there may be more heterogeneity in the pre-pairing population than we account for here, with some mouse pregnancy outcomes predictable even before pairing. The apparent pregnancies that prematurely terminate do so after a variable number of days (data not shown).

**Fig 4:**
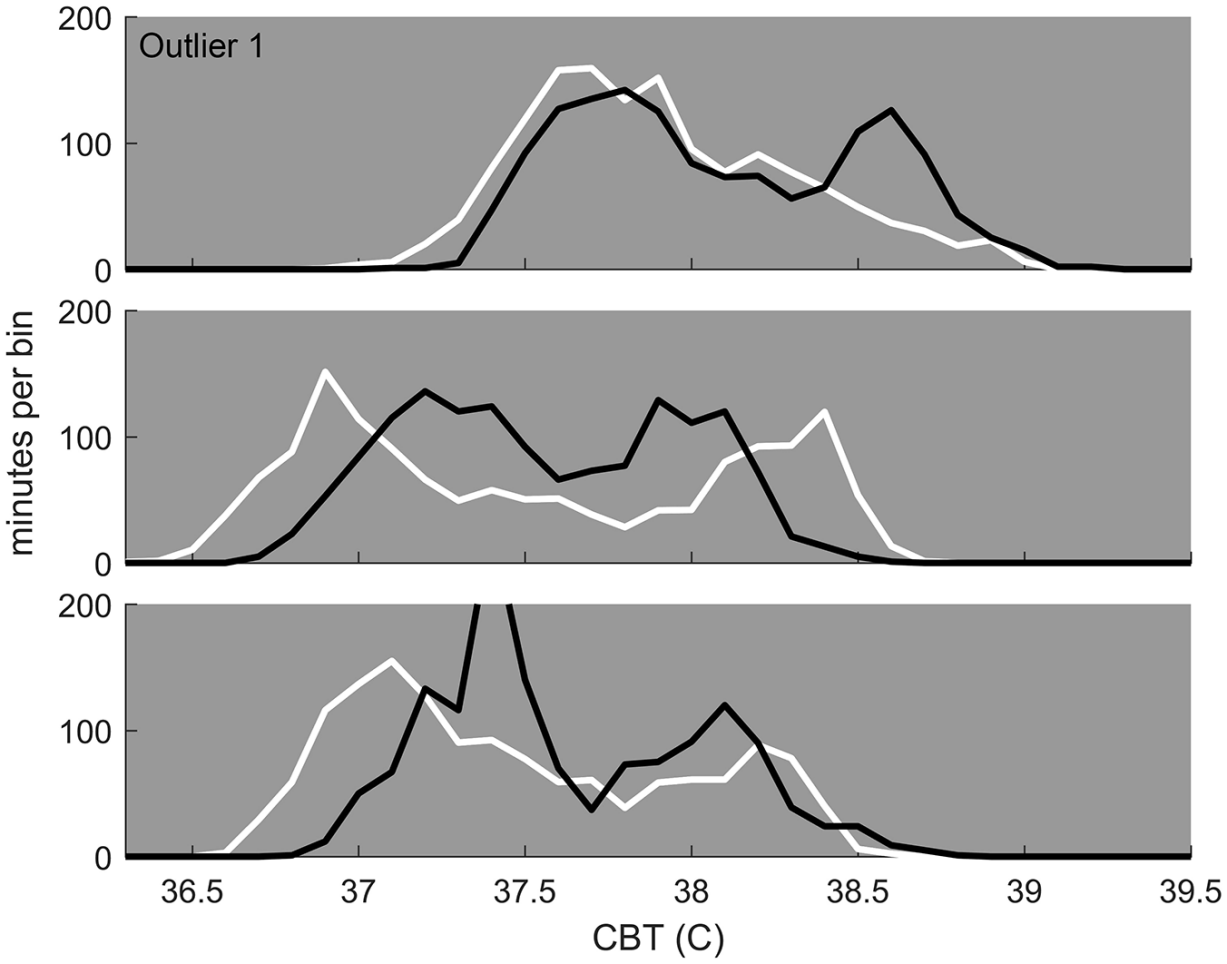
Within-animal comparison of pregnancies. Three mice were paired twice, and each had one successful pregnancy (white) and one apparent pregnancy that did not come to term (black). Histogram of temperature on the second day post pairing, as in Fig 3D, shows that the trend seen across animals—a relative increase in the number of relatively cold min for pregnancies that will come to term—holds within mice as well: all three individuals show an increased amount of time relatively cold in the pregnancy that came to term compared to the one that did not for the same animal. One of these mice was one of the aberrantly warm outliers (top), and so compared to the population at large was one of the few for which the pregnancy-outcome prediction failed, illustrating that within animal comparisons, and comparison to mice showing similar temperature profiles, may allow increased accuracy of future predictions.

Three mice had one successful pregnancy and one apparent pregnancy which terminated prematurely. In all three cases, the pregnancy that came to term showed a higher frequency of “cold” (left-tail) min following pairing than the unsuccessful pregnancy (Fig 4), consistent with the pattern seen across individuals in Fig 3. The intra-individual pattern appears to match the population pattern of successful pregnancies: a relatively left-shifted left-tail of temperature distributions. One of these three mice was also one of the high-temperature outliers (Fig 4A), but despite its different baseline temperature distribution, the relationship of relatively higher frequency low temperatures and pregnancy success appears be maintained across this different CBT phenotype (though this is only a single observation, and other CBT phenotypes may show different properties).

**Fig 5:**
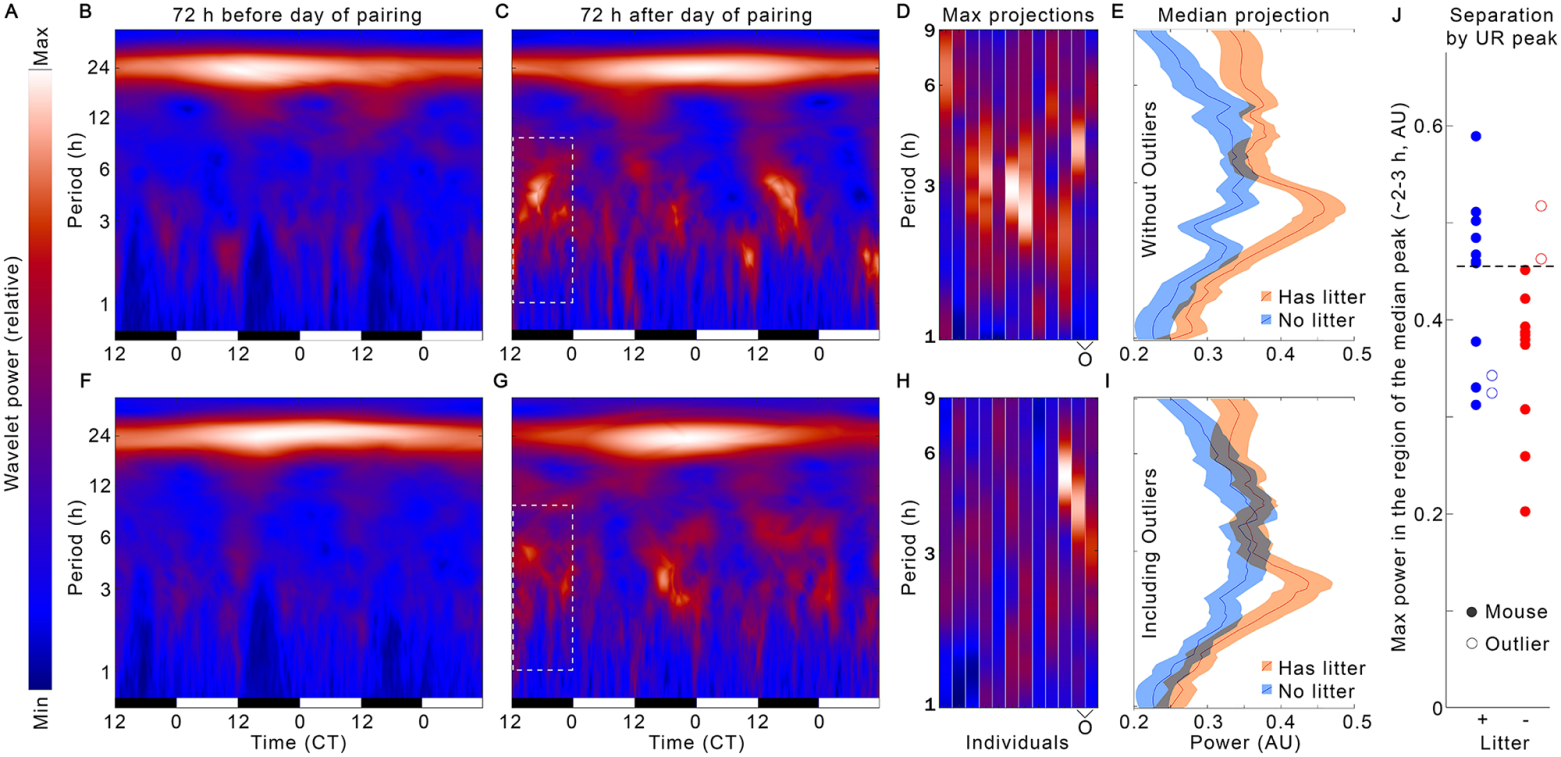
Wavelet analysis reveals differences within 12 h after the day of pairing indicative of successful pregnancies. Median wavelet transformations of CBT from all mice, including outliers, for the 3 d before pairing (B, F) and 3 d after pairing (C, G), with time along the X axis (light:dark bars indicate light cycle), period along the Y axis, and increases in power per time per period noted by shift in color from blue to red to white (color map in A). Dotted box (C, G) highlights 12 h region of increased ultradian power in mice that show a post-pairing rise in basal CBT and later come to term (top row, B, C, D, whereas the bottom row [F, G, H] displays those individuals that show an initial elevation in CBT after pairing, but which did not deliver a litter). Each individual’s profile of this boxed region is shown as a 2D maximum-intensity projection (D, H) to illustrate the individual variance, with the 4 outliers, noted previously, highlighted with an “O” on the X axis. Median +/- standard error of the spectral power profiles of these projections (E, I) reveals a significant, broad increase in power with a peak around 3 h periodicity for the mice that came to term (orange) as compared to those that did not (blue). This effect is significant whether outliers are excluded (E, χ^2^ = 363.2, *p* = 5.64x10^-81^) or included (I, χ^2^ = 128.15, *p* = 1.04x10^-29^). Individuals’ max power in the region of the ~3h peak (J) indicates that a majority of mice can be successfully separated by even this highly simplified metric (*p* = 0.026; dotted line indicates threshold above which a majority of successful pregnancies appear, and no unsuccessful, non-outlier pregnancies appear). Note that outliers from each group appear in the range of the opposite group, highlighting the importance of identifying heterogeneity to improve accuracy.

Separation of ultimately successful pregnancies from ultimately terminated or apparent pregnancies can be achieved within the first 12 h after the day of pairing using frequency analysis by high-resolution time. Analysis of CBT frequency composition at 1-min intervals by wavelets reveals a significant power increase specifically in ultradian CBT frequencies in the first 12 h after the day of pairing in pregnancies that come to term (Fig 5B, 5C and 5D) relative to those apparent pregnancies that do not (Fig 5F, 5G and 5H). This pattern can be seen more easily in 2-dimensional maximum-intensity projections of the boxed region for each individual in (Fig 5D and 5H). The projections from each group show significantly different spectral power distributions even with outliers included (Fig 5I; χ^2^ = 128.15, *p* = 1.04x10^-29^), and this pattern is enhanced when outliers are removed (Fig 5E; χ^2^ = 363.2, *p* = 5.64x10^-81^). The projections reveal a broad increase in power across frequencies in pregnancies that will come to term, with a large peak at ~ 3 h periodicity in successful pregnancies. Further simplification to the maximum value of this peak (Fig 5J) illustrates that the majority of individuals can be separated based on even this single point measure.

## Discussion

Continuous monitoring of CBT allows for rapid detection of pregnancy following mating with 100% accuracy, with all eventual births confirmed after detection of CBT changes in the first rest phase after pairing. Other pregnancy-like events are detectable as similar CBT changes but that revert to patterns present during estrous cycle rhythms before completing the pregnancy (with no litter being produced). Such events can be detected by the same continuous CBT monitoring approach—occurrences that would go undetected by normal observation. Unsuccessful pregnancy-like events can be distinguished from pregnancies that will come to term within 48 h from the day of pairing by daily frequency analysis (frequency distribution of CBT over days), and within 12 h after the day of pairing by frequency analysis by time, with wavelet transformations of 1-min CBT data revealing both a broad increase in power, and a specific peak of increased power around 3 h periodicity. This rapid detection and classification without disturbing animals, is to our knowledge, unprecedented. It highlights that the use of high temporal resolution recordings—measurements increasingly possible as sensor size and price continually decrease, is likely an approach that can be applied beyond pregnancy detection.

We focused on CBT as opposed to the more commonly-tracked modality of LA. We find that on average, LA also shows daily structural changes with pregnancy onset and progression, but LA signals were sufficiently variable for individual mice to preclude detection with the accuracy, speed, or discrimination achieved with CBT. Because CBT is correlated with LA, this difference is somewhat surprising. However, unlike LA, CBT is coupled to many hormonal systems [8,9,16–18], and may provide physiological information unavailable in LA. Additionally, CBT never reports with a zero value, as LA does at rest, making rest-phase change more apparent through CBT than LA. More sophisticated analyses of LA might reveal equally useful information, as might combined analyses of LA and CBT.

We did not confirm pregnancy by measures other than CBT and LA. No hormone assays were performed, and uteri were not collected, because mice were part of an ongoing breeding program. The observation that the apparent pregnancies that terminated prematurely did so after a variable range of days across individuals suggests that they were not a single, stereotyped pseudopregnancy, or if they are, that pseudopregnancies themselves are heterogeneous in manifestation, as opposed to stereotyped responses to vaginal stimulation [19]; this merits further investigation. It is likely that the changes in CBT frequency composition observed after pairing that are predictive of pregnancy outcome reflect a physiological response of the dam that occurs in advance of implantation on p5 [20]. This implies that the ultimate success of the pregnancy was strongly influenced by the state of the dam at pairing, or alternatively by her reaction to pairing. No obvious changes in power spectra are apparent before pairing, so it is unclear which physiological responses within the day of pairing may have affected pregnancy outcome. Ultradian rhythms expression changes within several hours from the day of pairing, and this observation deserves future attention. Whereas neither of the outcome prediction patterns we describe here succeeds with the same 100% accuracy as the pregnancy detection pattern, both allow identification for the majority of individuals. Whether unsuccessful individuals, primarily mice identified as outliers by their unusual pre-pregnancy temperature distributions, have different physiological responses, or responses translated into CBT differently, remains to be explored. Our approaches are not the ultimate pregnancy outcome predictions. Rather, these first analyses indicate that in most mice at least, pregnancy outcome is influenced by physiological changes that occur within hours of conception, a surprising and potentially widely useful finding. Refining which patterns or analyses carry the most information for which individuals will require further investigation.

The frequency analysis by day reveals what appears to be previously undescribed heterogeneity in the temperature regulation of mice. Three outliers appear to have substantially right-shifted CBT distributions both before, and especially after, pairing, highlighting the likelihood that high temporal resolution CBT may reveal phenotypic diversity that would otherwise go undetected, and not investigated. One outlier also appears after pairing, showing the largest CBT-frequency left-shift of any animal post-pairing, despite the pregnancy being one that did not come to term (whereas the relative left-shift of the left tail was the indicator of success in the other pregnancies). Expansion of high temporal resolution data gathering, to characterize “normal” responses, normal heterogeneity, and corresponding physiological relevance can be of translational value to understand the heterogeneity of patterns across populations due to age, genetic background, diet, health, and season.

The frequency analysis by wavelets revealed that rhythmic changes in the ultradian range were most predictive about pregnancy outcome. The peak of this ultradian rhythm (UR) power was at ~ 3 h (Fig 4d). A number of physiological rhythms have periods in this range [21–24]. Human cortisol rhythms, with periods in this range exhibit a stable phase relationship with distal and core body temperature [25]. The mouse suprachiasmatic nucleus, central orchestrator of circadian rhythms in mammals, also displays URs in clock gene expression with a period of around 3 h [26] and behavioral arousal in mice displays URs of 3–4 h [27,28]. High temporal resolution recordings of CBT, which is affected by various hormonal and behavioral states [8,9,16–18], may be a useful tool to advance understanding of URs and predicting fertility and infertility in mice and other mammals, including livestock and humans.

## Supporting Information

S1 Code Matlab code used for all analysis and figure creation in this manuscript. (M)

S1 Dataset All data analyzed in this manuscript. (XLSX)
